# ^99m^Tc-sestamibi and ^18^F-fluorodeoxyglucose imaging in patients with cardiogenic shock: A pilot study

**DOI:** 10.3389/fcvm.2022.1047577

**Published:** 2022-11-08

**Authors:** Cuncun Hua, Qizhe Cai, Xiao-Ying Xi, Mingming Lin, Li Wang, Lina Li, Dandan Yao, Xiaoyan Liu, Lei Zhao, Lefeng Wang, Pixiong Su, Boqia Xie

**Affiliations:** ^1^Cardiac Center, Beijing Chaoyang Hospital, Capital Medical University, Beijing, China; ^2^Department of Echocardiography, Beijing Chaoyang Hospital, Capital Medical University, Beijing, China; ^3^Department of Nuclear Medicine, Beijing Chaoyang Hospital, Capital Medical University, Beijing, China

**Keywords:** cardiogenic shock, ST-segment elevation myocardial infarction, perfusion, fluorodeoxyglucose, major adverse cardiac events

## Abstract

**Background:**

Whether perfusion/metabolism imaging differs between matched ST-segment elevation myocardial infarction (STEMI) patients with and without cardiogenic shock (CS) remains unknown.

**Methods:**

Seventeen STEMI patients with CS (13 men, 60 ± 12 years) and 16 matched STEMI patients without CS (15 men, 54 ± 15 years) were prospectively recruited. All patients underwent baseline ^99m^Tc-sestamibi/^18^F-fluorodeoxyglucose (FDG) imaging and echocardiography 6 ± 2 days post-infarction. Nine patients with CS and seven without CS had repeated imaging 98 ± 7 days post-infarction. The total perfusion deficit (TPD) and total FDG uptake deficit (TFD) were calculated to assess the percentages of impaired perfusion and metabolism over the left ventricle. Patients were followed up for 337 days (213–505 days) and the major adverse cardiac events (MACE) were recorded.

**Results:**

TPD was greater in patient with CS and was independently related to the presence of CS (OR: 4.36, *p* = 0.013). Both acute- and convalescent TFD were inversely related to the improvement ratio of LVEF (*r*-values: −0.62, −0.73; both *p* < 0.05). MACE occurred in 16 patients (10 CS and 6 non-CS), and acute TFD was predictive of MACE in those with CS (HR: 2.06, *p* = 0.038).

**Conclusion:**

In this pilot study, we demonstrated that STEMI patients with CS had a significantly increased TPD, which was relevant to the presence of CS. Acute TFD was associated with improvement in LVEF, and was predictive of MACE in patients with CS.

## Graphical Abstract

TPD was increased in STEMI patients with CS and was relevant to the presence of CS. Acute TFD was associated with improvement in LVEF, and was predictive of MACE in patients with CS. STEMI: ST-segment elevation myocardial infarction; CS, cardiogenic shock; TPD, Total perfusion deficit; TFD, total FDG uptake deficit; LVEF, left ventricular ejection fraction; MACE, major adverse cardiac events. 
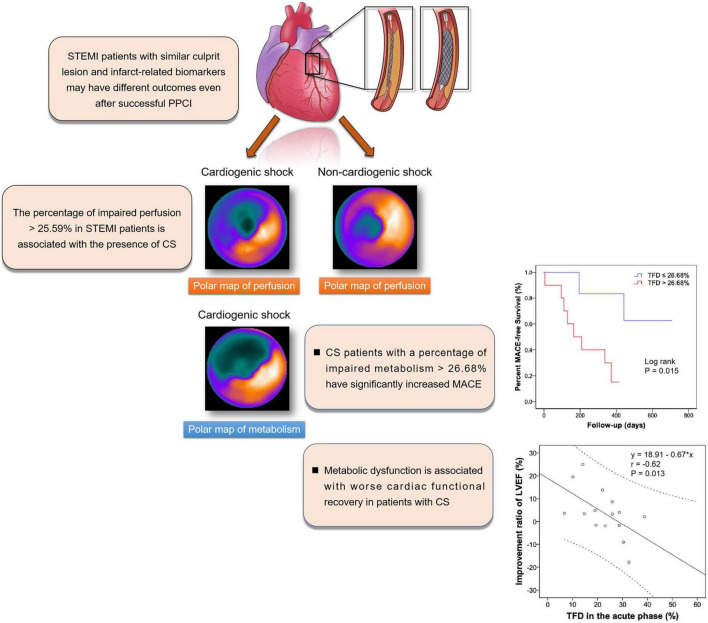

## Introduction

Cardiogenic shock (CS) remains a high-risk subset of patients with ST-segment elevation myocardial infarction (STEMI). Despite significant improvements in the management of STEMI in recent decades, the prevalence of CS has remained unchanged (1). Several risk prediction models for short-term mortality in CS have been proposed, including the CardShock risk score, the IABP-SHOCK II risk score, and the CS4P model (2–4). However, these models mainly rely on clinical awareness and serum biomarkers, with a lack of indicators that directly reflect the severity of impaired myocardial perfusion and metabolism–two parameters that are related to cardiac function and long-term prognosis in STEMI (5, 6).

^99m^Tc-sestamibi single photon emission computed tomography (SPECT) myocardial perfusion imaging combined with fasting ^18^F-fluorodeoxyglucose (FDG) positron emission tomography/computerized tomography (PET/CT) is a well-established protocol for assessing myocardial perfusion and metabolism (7). Nevertheless, no previous studies have explored the clinical value of this modality in patients with CS. And it remains unknown whether perfusion/metabolism imaging differs between matched STEMI patients with and without CS.

Therefore, in this prospective study, we compared ^99m^Tc-sestamibi/FDG imaging in matched STEMI patients with- and without CS after successful primary percutaneous coronary intervention (PPCI) with a TIMI 3 flow, and hypothesized that myocardial perfusion and metabolism impairment may be different in matched patients with- and without CS, and sought to investigate their predictive value for major adverse cardiac events (MACE) in patients with CS.

## Materials and methods

### Study population

This prospective study was approved by the Chaoyang Hospital Ethics Committee and was conducted in agreement with the Declaration of Helsinki. The trial was registered at Chictr.org.cn (No. ChiCTR2000034052). From June 2020 to July 2021, eligible patients admitted to the cardiac care unit (CCU) of Beijing Chaoyang Hospital with first time STEMI were initially enrolled after successful PPCI (TIMI 3 flow) within 12 h of symptom onset. Additional inclusion criteria included single-vessel disease [only one major vessel (≥ 2 mm in diameter) with > 70% stenosis] involving the proximal anterior descending artery (LAD). The exclusion criteria were as follows: < 18 or > 80 years of age; previous cardiac events, cardiomyopathy, or congenital heart disease; massive pulmonary embolism; shock without a cardiogenic cause; development of mechanical complications; history of abnormal renal function; or other severe concomitant diseases associated with a life expectancy of ≤ 6 months.

The diagnosis for CS included (8, 9): systolic blood pressure < 90 mmHg for > 30 min or requirement of catecholamine infusion to maintain a systolic pressure of > 90 mmHg; clinical signs of pulmonary congestion; or impaired end-organ perfusion including at least one of the following: altered mental status; cold, clammy skin and limb; oliguria with urine output <30 ml/h; or arterial lactate level > 2.0 mmol/L.

The CS and non-CS group participants were matched for culprit vessel, age, sex, body mass index (BMI), comorbidities, risk factors, and maximum levels of creatine kinase-MB (CK-MB), and troponin I (cTnI).

### ^99m^Tc-sestamibi single photon emission computed tomography and ^18^F-fluorodeoxyglucose positron emission tomography/computerized tomography imaging

After 12 h fasting, perfusion images were collected using a dual-head SPECT/CT scanner (infinia hawkeye 4, GE, USA), and FDG positron emission tomography/computerized tomography (PET/CT) images were obtained with a 16-slice PET/CT scanner (Discovery STE, GE, USA) (Supplementary Methods 1, 2). Both SPECT and PET/CT data were analyzed in an automated manner (QPS: version 3.1, Cedars-Sinai Medical Center, Los Angeles, CA, USA) to calculate the percentages of impaired perfusion (total perfusion deficit [TPD]) and metabolism (total FDG uptake deficit [TFD]) over the left ventricle.

Bone marrow and spleen are the major sources and reservoirs of inflammatory monocytes/macrophages, and their FDG activities were calculated to reflect the systemic inflammation (Supplementary Method 3) (10).

### Echocardiography

Echocardiographic examination was performed on the same day as ^99m^Tc-sestamibi/FDG imaging. Images were acquired by one experienced echocardiographic investigator on a Vivid E95 ultrasound instrument (Vingmed Ultrasound, Horten, Norway) with M5Sc and 4V multiphase-array transducers. The left ventricular end-diastolic diameter (LVEDD), left ventricular end-systolic diameter (LVESD), and LVEF were measured using the Biplane Simpson’s method on apical four- and two-chamber view.

### Blood analysis

At admission, all patients underwent basic complete blood count, metabolic panel [cTnI, BNP, high-sensitive C-reactive protein (hsCRP), cholesterol level, triglyceride level, liver function test, renal function test, electrolytes], and arterial blood gas (including lactate) investigations. Following which, serial blood samples were tested in the CCU before patients were transferred to the ordinary ward.

### Follow-up

All patients were regularly followed at the clinic. The primary endpoint was the incidence of MACE, which was defined as a composite of cardiovascular death, recurrent myocardial infarction, rehospitalization for revascularization or unstable angina, congestive heart failure, and stroke. Repeat myocardial ^99m^Tc-sestamibi SPECT, FDG PET/CT imaging, and echocardiography were performed at 3 months post-infarction.

### Statistical analysis

SPSS Statistics (Version 24; IBM) was used to perform the statistical analyses. Continuous variables are described as mean (SD) or medians (interquartile ranges). Categorical variables are expressed as absolute numbers and percentages. Variables between groups were compared using Student’s *t*-test, the Mann–Whitney *U*-test, the chi–square test, or the Fisher’s exact test, depending on the parametric nature of the data. Logistic and Cox regression analyses were employed to explore the relevant factors for the presence of CS and the predictors of MACE in patients with CS. A receiver operating characteristic curve (ROC) was generated to determine the prognostic value of TFD. A Kaplan–Meier plot was used to characterize the cumulative mortality during the follow-up, and the log-rank test was used for the comparison between groups. Two published risk prediction models for short-term mortality in CS were employed: the CardShock risk score and the IABP-SHOCK II risk score. Net reclassification improvement (NRI) and integrated discrimination improvement (IDI) statistical analyses were performed using R software (Version 4.2.0)^1^ to evaluate the ability of the new predictive model. A *p*-value < 0.05 was considered statistically significant.

## Results

The patients’ baseline characteristics are presented in Table 1. Seventeen patients with CS (13 men, 60 ± 12 years) and 16 patients without CS (15 men, 54 ± 15 years) were prospectively recruited. All patients underwent baseline ^99m^Tc-sestamibi/FDG imaging 6 ± 2 days post-MI.

**TABLE 1:** Baseline characteristics.

	CS (*n* = 17)	Non-CS (*n* = 16)	*P*-value
Age, years	60 ± 12	54 ± 15	0.21
Male, *n* (%)	13 (76.47)	15 (93.75)	0.34
BMI, kg/m^2^	25.41 ± 3.22	27.22 ± 4.40	0.18
Hypertension, *n* (%)	6 (35.29)	7 (43.75)	0.73
Diabetes mellitus, *n* (%)	2 (11.76)	1 (6.25)	1.00
Stroke, *n* (%)	3 (17.65)	1 (6.25)	0.60
Hyperlipidemia, *n* (%)	6 (35.29)	6 (37.50)	1.00
Active smoking, *n* (%)	7 (41.18)	11 (68.75)	0.17
Family history, *n* (%)	1 (5.88)	1 (6.25)	1.00
**CS SCAI classification**			
Stage A (at risk)	2 (11.80)	N/A	N/A
Stage B (beginning)	7 (41.20)	N/A	N/A
Stage C (classic)	8 (47)	N/A	N/A
Stage D (deteriorating)	0	N/A	N/A
Stage E (extremis)	0	N/A	N/A
Pain to balloon (h)	2.64 ± 2.75	2.61 ± 2.91	0.98
**PCI procedures**			
Complex stenting techniques	3 (17.65)	5 (31.25)	0.44
Aspiration thrombectomy	3 (17.65)	6 (37.5)	0.26
Tirofiban	9 (52.94)	10 (62.5)	0.73
**Serum biomarkers**			
cTnI_*max*,_ ng/ml	294.44 ± 145.71	251.69 ± 132.45	0.39
CKMB_*max*,_ ng/ml	268.33 ± 152.57	213.26 ± 133.81	0.25
Lactate, mmol/L	2.45 ± 0.55	2.06 ± 1.00	0.23
Creatinine_*max*_, μmol/L	69.48 ± 19.73	69.52 ± 10.25	0.99
hsCRP_*max*_, mg/L	22.18 ± 18.64	9.27 ± 8.01	**0.028**
WBC_*max*_, 10^9^/L	12.88 ± 4.48	12.48 ± 2.35	0.75
LDH_*max*_, U/L	1035.47 ± 448.93	824.38 ± 419.09	0.17
BNP_*max*_, pg/ml	491.24 ± 218.37	185.63 ± 113.31	**<0.001**
Fasting blood glucose, mmol/L	6.11 ± 0.78	6.26 ± 1.59	0.74
**Risk prediction scores**			
CardShock risk score	2 (2, 2.5)	0.5 (0, 1)	**<0.001**
IABP-SHOCK II risk score	0 (0, 1)	0 (0, 0.75)	0.41
Length of stay in CCU, days	5.88 ± 1.87	3.88 ± 0.81	**<0.001**
**Echocardiography**			
LVEF,%	53.71 ± 8.88	59.53 ± 5.11	**0.03**
LVEDD, mm	46.41 ± 3.10	49.13 ± 3.40	**0.03**
LVESD, mm	30.06 ± 3.73	29.73 ± 4.92	0.83
**Nuclear imaging**			
TPD,%	26.63 ± 12.30	17.89 ± 10.21	**0.038**
TFD,%	26.19 ± 10.95	20.77 ± 7.52	0.11
SUVspleen	142.35 ± 51.97	104.19 ± 42.17	**0.03**
SUVbone-marrow	151.94 ± 29.38	124.50 ± 37.74	**0.03**

CS, cardiogenic shock; BMI, body mass index; cTnI, troponin I; CKMB, creatine kinase-MB; hsCRP, hypersensitive C reactive protein; WBC, white blood cell; LDH, lactic dehydrogenase; BNP, B type natriuretic peptide; LVEF, left ventricular ejection fraction; LVEDD, left ventricular end diastolic diameter; LVESD, left ventricular end systolic diameter; TPD, Total perfusion deficit; TFD, total FDG uptake deficit; SUV, standardized uptake value.

The bold values indicate a *p*-value < 0.05.

**FIGURE 1:**
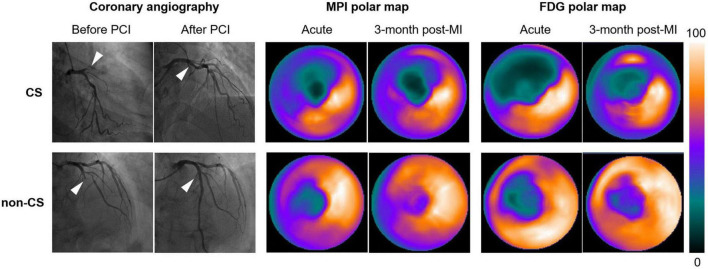
Representative ^99m^Tc-sestamibi/FDG images of STEMI patients with and without CS following successful PPCI in the LAD (white arrows). Polar maps identified larger extent of impaired myocardial perfusion and metabolism in patients with CS both in the acute phase and in the convalescent phase. CS, cardiogenic shock; PPCI, primary percutaneous coronary intervention; LAD, left anterior descending artery; TFD, total FDG uptake deficit.

According to the SCAI shock classification, in the CS group, two patients were in stage A, seven patients in stage B, and eight patients in stage C. There were no significant differences in the demographics, comorbidities, risk factors, pain-to-balloon time, PCI procedures or maximum levels of cTnI and CKMB, between patients with and without CS (Table 1, all *p* > 0.05). Nevertheless, patients with CS had increased TPD (Figure 1), lower LVEF, higher BNPmax, hs-CRPmax, standardized uptake values of spleen and bone marrow (SUVspleen, SUVbone-marrow), and greater CardShock risk scores (all *p* < 0.05). Comparison between TPD and TFD yielded no statistical difference in both CS and non-CS arms (all *p* > 0.05). TPD was inversely related to LVEF in both patients with CS (*r* = −0.66, *p* = 0.004) and without CS (*r* = −0.66, *p* = 0.01). Multivariate regression analysis demonstrated that TPD [odds ratio (OR): 4.36, 95% confidence interval (CI):1.37–13.90, *p* = 0.013] and hs-CRP (OR: 1.15, 95%CI: 1.02–1.30, *p* = 0.02) were independently related to the presence of CS (Table 2). The cutoff value of TPD was set as 25.59% according to the ROC curve [area under the curve (AUC): 0.73; sensitivity 71%, specificity 80%].

**TABLE 2:** Univariate and multivariate regression analysis for the presence of cardiogenic shock.

	Univariate analysis			Multivariate analysis		
	OR	95% CI	*P*-value	OR	95% CI	*P*-value
**hsCRP_*max*_, mg/L**	1.09	1.004–1.18	0.04	1.15	1.02–1.30	**0.02**
**TPD, % (per 10% increase)**	2.00	1.004–3.97	**0.049**	4.36	1.37–13.90	**0.013**

hsCRP, hypersensitive C reactive protein; TPD, total perfusion deficit; OR, odds ratio; CI, confidence interval.

The bold values indicate a *p*-value < 0.05.

**FIGURE 2:**
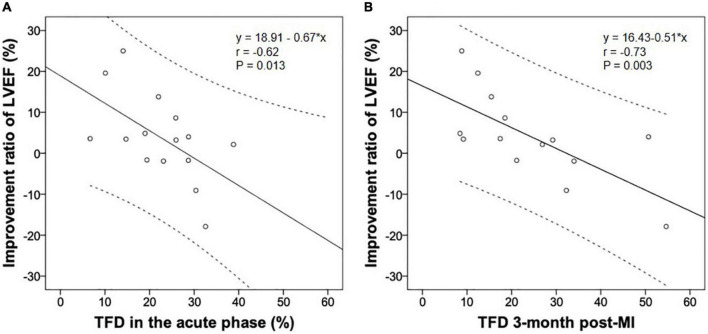
The improvement ratio of LVEF post-infarction was inversely associated with the TFD in the acute **(A)** and convalescent phase **(B)**. LVEF, left ventricular ejection fraction; MI, myocardial infarction; TFD, total FDG uptake deficit.

Nine patients with CS and seven without had repeat imaging 98 ± 7 days post-infarction. Comparisons of the imaging data between acute and convalescent phases (Table 3) demonstrated the following: systemic inflammation (indicated by spleen and bone marrow activity) was alleviated in patients with and without CS (both *p* < 0.05); LVEDD and LVESD significantly increased in patients with CS (both *p* < 0.05); and the improvement ratio of LVEF was inversely correlated with acute and convalescent TFD (*r*-values: −0.62 and −0.73, *p*-values: 0.013 and 0.003, respectively) (Figure 2).

**TABLE 3:** Comparisons between the acute and convalescent (3-month post myocardial infarction) imaging in patients with- and without cardiogenic shock.

	CS (*n* = 9)			Non-CS (*n* = 7)		
	Acute	Convalescent	*P*-value	Acute	Convalescent	*P*-value
**Echocardiography**						
LVEF, %	53.00 ± 7.26	53.11 ± 6.53	0.96	58.83 ± 3.82	63.33 ± 2.66*	0.08
LVEDD, mm	48.22 ± 3.19	51.67 ± 3.74	**0.009**	47.83 ± 2.64	50 ± 2.76	0.27
LVESD, mm	30.33 ± 4.77	34.33 ± 5.85	**0.04**	29.83 ± 3.71	30.83 ± 2.71	0.63
**Nuclear imaging**						
TPD,%	29.71 ± 9.86	23.89 ± 11.63	0.08	19.58 ± 8.05	16.10 ± 8.28	0.24
TFD,%	24.99 ± 10.41	29.79 ± 14.76	0.29	19.14 ± 4.99	14.22 ± 8.87	0.10
SUVspleen	150.50 ± 59.33	75.38 ± 23.21	**0.001**	122.83 ± 51.55	89.33 ± 39.85	0.06
SUVbone-marrow	152.33 ± 37.06	87.44 ± 13.37	**0.003**	106.83 ± 18.33	80.33 ± 19.29	**0.005**

*p < 0.05 compared with the convalescent imaging in patients with CS. LVEF, left ventricular ejection fraction; LVEDD, left ventricular end diastolic diameter; LVESD, left ventricular end systolic diameter; TPD, Total perfusion deficit; TFD, total FDG uptake deficit; SUV, standardized uptake value.

The bold values indicate a *p*-value < 0.05.

**FIGURE 3:**
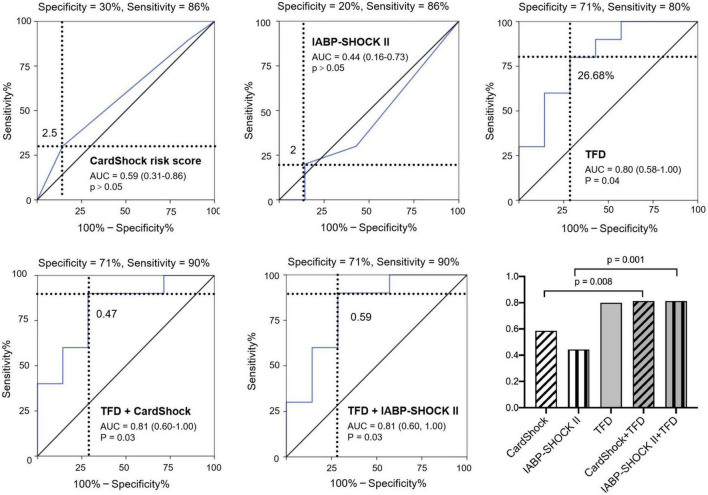
Predictive value of post-infarction MACE in patients with CS using CardShock risk score, IABP-SHOCK II risk score, TFD, combination of CardShock risk score and TFD, and combination of IABP-SHOCK II risk score and TFD. TFD added significant predictive information to these models. AUC: area under the receiver-operating characteristic curve; TFD, total FDG uptake deficit; MI, myocardial infarction; MACE, major adverse cardiac events; CS, cardiogenic shock.

**FIGURE 4:**
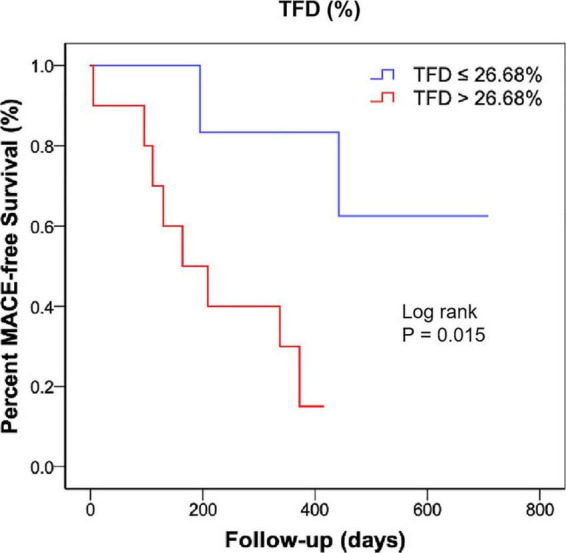
Kaplan-Meier curve showing MACE-free survival rate according to the TFD in patients with CS. MACE, major adverse cardiac events; TFD, total FDG uptake deficit; CS, cardiogenic shock.

All patients received optimal guideline-directed medical therapy (Table 4) and were regularly followed up for 337 (213–505) days. Sixteen patients (10 with CS and 6 without CS) had MACE. Among the patients who had repeat imaging, no statistical differences were found regarding the medical therapy (Table 5). Multivariate Cox regression analysis determined that acute TFD was independently predictive of MACE in patients with CS [hazard ratio (HR): 2.06, 95%CI: 1.04–4.08, *p* = 0.038] (Table 6). The cutoff value of TFD was set as 26.68% according to the ROC curve (AUC: 0.80; sensitivity 80%, specificity 71%) (Figure 3), and the log-rank test showed a significant difference in survival between CS patients with high and low TFD (mean MACE: 223 vs. 566 days; 95% CI: 138–309 vs. 404–729; *p* = 0.015) (Figure 4).

**TABLE 4:** Comparisons of the medical therapy between patients with- and without CS.

	CS (*n* = 17)	Non-CS (*n* = 16)	*P*-value
Aspirin	17 (100)	16 (100)	N/A
P2Y12 inhibitor	17 (100)	16 (100)	N/A
Statin	17 (100)	14 (87.5)	0.23
β-blocker	15 (88.24)	12 (75)	0.40
ACEI/ARB	5 (29.41)	13 (81.25)	0.005
ARNI	9 (52.94)	0 (0)	0.001
MRA	5 (29.41)	0 (0)	0.044
SGLT2i	1 (5.88)	1 (6.25)	N/A

ACEI/ARB, angiotensin converting enzyme inhibitor or angiotensin receptor blocker; ARNI, angiotensin receptor-neprilysin inhibitor; MRA, mineralocorticoid receptor antagonists; SGLT2i, sodium glucose co-transporter 2 inhibitor.

**TABLE 5:** Comparisons of the medical therapy between patients with- and without CS who had repeat imaging.

	CS (*n* = 9)	Non-CS (*n* = 7)	*P*-value
Aspirin	9 (100)	7 (100)	N/A
P2Y12 inhibitor	9 (100)	7 (100)	N/A
Statin	9 (100)	7 (100)	N/A
β-blocker	8 (88.89)	4 (57.14)	0.26
ACEI/ARB	2 (22.22)	4 (57.14)	0.30
ARNI	4 (44.44)	0	0.09
MRA	3 (33.33)	0	0.21
SGLT2i	1 (11.11)	0	1

ACEI/ARB, angiotensin converting enzyme inhibitor or angiotensin receptor blocker; ARNI, angiotensin receptor-neprilysin inhibitor; MRA, mineralocorticoid receptor antagonists; SGLT2i, sodium glucose co-transporter 2 inhibitor.

**TABLE 6:** Cox regression analysis for MACE in patients with cardiogenic shock.

	Univariate analysis			Multivariate analysis		
	HR	95% CI	*P*-value	HR	95% CI	*P*-value
**TFD,% (per 10% increase)**	2.06	1.04–4.08	**0.038**	2.06	1.04–4.08	**0.038**
**LVESD, mm**	1.16	0.96–1.40	0.13	1.06	0.82–1.38	0.65
**SUVspleen**	0.99	0.98–1.004	0.16	0.99	0.97–1.008	0.22

MACE, major adverse cardiac events; HR, Hazard ratio; CI, confidence interval; TFD, total FDG uptake deficit; LVESD, left ventricular end systolic diameter; SUV, standardized uptake value.

The bold values indicate a *p*-value < 0.05.

Models of CardShock and IABP-SHOCK II risk score had an AUC of 0.59 and 0.44, respectively, for predicting MACE in patients with CS. Moreover, incorporating TFD into these models significantly improved patient reclassification and discrimination (Table 7 and Figure 3).

**TABLE 7:** Risk prediction models.

	AUC	*P*-value	NRI (95%CI)	*P*-value	IDI (95%CI)	*P*-value
CardShock risk score	0.59	0.56	N/A	N/A	N/A	N/A
IABP-SHOCK II risk score	0.44	0.70	N/A	N/A	N/A	N/A
TFD	0.80	**0.04**	N/A	N/A	N/A	N/A
CardShock risk score + TFD	0.81	**0.03**	1.03 (0.20–1.86)	**0.02**	0.30 (0.04–0.55)	**0.02**
IABP-SHOCK II risk score + TFD	0.81	**0.03**	1.23 (0.46–1.99)	**0.002**	0.31 (0.05–0.56)	**0.02**

AUC, area under the curve; NRI, net reclassification index; CI, confidence interval; IDI, integrated discrimination improvement; TFD, total FDG uptake deficit.

The bold values indicate a *p*-value < 0.05.

## Discussion

To our knowledge, this is the first study to explore the clinical value of ^99m^Tc-sestamibi/FDG imaging in STEMI patients with CS. Our results demonstrated that (1) TPD in the acute phase was an independent factor for the presence of CS; (2) TFD in the acute and convalescent phases were inversely associated with the improvement ratio of LVEF; (3) TFD in the acute phase was independently predictive of MACE in patients with CS; and (4) integration of the TFD into the CardShock and IABP-SHOCK II risk models significantly improved the prediction power for MACE in patients with CS.

The occurrence of CS remains high in patients with STEMI even in the PPCI era. Although the primary aim in STEMI is early restoration of patency and antegrade reflow, downstream myocardial reperfusion is not guaranteed (11). In this study, even though the STEMI patients were well-matched between the CS and non-CS groups, more severe myocardial perfusion impairment was demonstrated in the CS arm, which was independently related to the presence of CS. As impaired myocardial reperfusion post-infarction has been reported to be associated with an increased rate of cardiac dysfunction, complications, and poor survival (12), we propose that the objective of STEMI management should be extended to the achievement of adequate reperfusion at the myocardial tissue level to reduce the incidence of CS, with close and appropriate monitoring of antithrombotic therapies to safely balance the increased risk of bleeding and thrombosis.

However, even reperfused myocardium often displays a prolonged mechanical dysfunction which is paralleled by sustained metabolic abnormalities under experimental and clinical conditions (13–15). Using ^11^C palmitic acid and ^18^F-FDG as tracers of fatty acid and glucose metabolism, Schwaiger et al. (13) reported suppressed fatty acid utilization and enhanced glucose uptake in reperfused myocardium. Moreover, in the isolated rat heart, Taegtmeyer et al. (14) reported that the myocardial ATP content and glycogen store remained low even after effective reperfusion. Furthermore, comparing perfusion/FDG imaging before and post-CABG, Tamaki et al. (15) observed significantly reduced FDG uptake in most reperfused myocardium, which was associated with impaired contraction. Consistent with these reports, impaired FDG uptake was observed in patients with STEMI in the current study, which was increased in the CS arm, although not significantly. As the TFD was inversely associated with LVEF improvement, treatment aimed at ameliorating myocardial metabolism dysfunction should be emphasized, for example, early application of trimetazidine.

According to the SCAI shock stage classification (16), most of our patients were in the “Beginning” and “Classic” stages, with a short-term mortality of < 12.4%. However, it is important to note that even these “non-high-risk” CS patients accounted for most of the cases of MACE during long-term follow-up. This is important because it indicates that a large number of events could originate in the “non-high-risk” CS population.

Until now, no risk prediction model has been developed for long-term MACE in patients with CS. Both the CardShock risk score and IABP-SHOCK II risk score were designed for short-term mortality, with a small AUC for predicting MACE in the current study. By incorporating perfusion and metabolic information into the regression model, we found that TFD was an excellent predictor of MACE in patients with CS. Moreover, a significant improvement in reclassification and discrimination was observed by integrating the TFD into the above risk models, suggesting the importance of metabolic assessment in the prognosis of CS.

### Limitations

This study has several limitations. First, the sample size was small, and most patients with CS were in stages A to C according to the SCAI classification, and patients with high short-term mortality were not included. Second, although patients with and without CS patients were matched by baseline characteristics, individual variance cannot be completely ruled out. Third, only patients with proximal LAD STEMI were recruited in the present study, future research including patients with diverse coronary lesions should be performed to extend and complement our findings. Therefore, a multicentered cohort study with a large sample size, including “high-risk” CS patients and a prolonged follow-up period is needed to validate our findings.

## Conclusion

In this pilot study, STEMI patients with CS had a significantly increased extent of perfusion impairment in the acute phase, which was associated with the presence of CS. The extent of reduced FDG uptake following infarction was inversely related to the improvement of LVEF, and was independently predictive of MACE in patients with CS.

## Data availability statement

The original contributions presented in this study are included in the article/Supplementary material, further inquiries can be directed to the corresponding author/s.

## Author contributions

CH and QC drafted the manuscript. CH, QC, X-YX, ML, LiW, LL, XL, and LZ collected clinical data and performed statistical analysis. X-YX, LiW, LL, and DY collected imaging data, performed analysis, and drafted the section “Materials and methods”. LeW, PS, and BX conceived the study and interpreted the results. All authors contributed to the article’s revision, agreed to its submission, had full access to original data, and participated in the study design.

## Footnotes

1
http://www.Rproject.org
