# Supplementary material for: 99mTc-sestamibi and 18F-fluorodeoxyglucose imaging in patients with cardiogenic shock: A pilot study
Source: Front Cardiovasc Med. 2022 Nov 8;9:1047577. doi: 10.3389/fcvm.2022.1047577 (PMC9678924; doi:10.3389/fcvm.2022.1047577)
Supplement: Supplementary file 1 [file Table_1.DOCX]

Supplementary Material

Table of contents

# Supplemental Methods

1. Myocardial ^99m^Tc-sestamibi single photon emission computed tomography (SPECT) acquisition
2. Myocardial ^18^F-fluorodeoxyglucose (FDG) positron emission tomography/computed tomography (PET/CT) acquisition
3. Spleen and Bone marrow FDG activities analysis

**Supplemental Methods**

**1. Myocardial ^99m^Tc-sestamibi SPECT acquisition**

Tomographic acquisition was performed was performed 40 minutes after intravenous injection of 740-925 MBq ^99m^Tc-sestamibi using a dual-head single photon emission computed tomography (SPECT)/CT scanner (infinia hawkeye 4, GE, USA) equipped with a low-energy, high-resolution collimator. The angle between the two detectors was 90°. Perfusion images were acquired with 60 views (25 s and 3° per view), with an acquisition matrix of 64 × 64 and a zoom factor of 1.30. The cardiac cycle was divided into 16 equal intervals. Images were reconstructed using a 3D-OSEM algorithm (10 subsets, 2 iterations), with attenuation and scatter correction.

**2. Myocardial ^18^F-FDG PET/CT acquisition**

After 12 hours of fasting, euglycemic hyperinsulinemic clamping preparation was performed according to a standard protocol. Oral glucose of 25-50 g was administered to the patients depending on their serum glucose level. In diabetes patients, acipimox was administrated (500 mg, oral dose) before glucose loading. Insulin was intravenously administrated if the blood glucose level > 9mmol/L at 45 minutes after oral glucose administration with close monitoring of blood glucose. ^18^F-FDG was intravenously injected when the blood glucose level reached 5.55 to 7.77mmol/L.

PET/CT imaging was performed 60 minutes after ^18^F-FDG (3.7 MBq/kg) injection when the blood glucose level reached 5.55 to 7.77mmol/L with a 16-slice PET/CT scanner (Discovery STE, GE, USA). CT parameters were 140 kV, 120 mA, pitch 1.375, 16 × 0.625 mm collimation, and section width of 5 mm. Two beds of PET images (5 minutes/bed, 3D mode) were acquired, with the heart set in the center of the view. Attenuation-corrected PET images (voxel size, 3.9 × 3.9 × 3.3 mm) were reconstituted from the CT date using a 3D ordered-subset expectation maximization (3D-OSEM) algorithm (14 subsets, 2 iterations).

**3. Spleen and Bone marrow FDG activities analysis**

Splenic activity was obtained by placing placing region of interest (ROI) guided by CT around the spleen on all transaxial sections. The average maximum standardized uptake value (SUVmax) values from all sections was recorded as the splenic activity. The activity of bone marrow was measured by drawing a ROI on a transaxial section of each vertebra from T-6 to T-8. The average SUVmax values of the three vertebrae was calculated to determine the activity of the bone marrow.
